# Electronic structure of organometal halide perovskite CH_3_NH_3_BiI_3_ and optical absorption extending to infrared region

**DOI:** 10.1038/srep37425

**Published:** 2016-11-18

**Authors:** H. X. Zhu, J.-M. Liu

**Affiliations:** 1 School of New Energy and Electronic Engineering, Yancheng Teachers University, Yancheng 224051, China; 2 Laboratory of Solid State Microstructures and Innovation Center of Advanced Microstructures, Nanjing University, Nanjing 210093, China

## Abstract

The electronic structure and optical absorption spectrum of organometal halide perovskite compound CH_3_NH_3_BiI_3_ as a substituting candidate of well-concerned CH_3_NH_3_PbI_3_ not only for environmental friendly consideration are studied using the first principles calculations. It is revealed that a Bi replacement of Pb in CH_3_NH_3_PbI_3_ does not change seriously the band edge structure but the bandgap becomes narrow. Consequently, CH_3_NH_3_BiI_3_ exhibits not only stronger visible light absorption than CH_3_NH_3_PbI_3_ does but more strong absorption in the infrared region, which is however absent in CH_3_NH_3_PbI_3_. It is suggested that CH_3_NH_3_BiI_3_ may be one of even more promising alternatives to CH_3_NH_3_PbI_3_ for spectrum-broad and highly-efficient solar cells.

Photovoltaic (PV) effect is an ideal photoelectric energy conversion process^1^,^2^ and has been one of the major mechanisms for green energy utilization in past decades, benefiting to human life substantially. The photoelectric conversion efficiency and cost-competitive manufacturing represent two major issues for PV researches and industry. At present, the photoelectric conversion efficiency of solar cells based on semiconducting materials such as Si and GaAs *et el* is between 12~25%^3^, driving sustained search for additional PV materials which are more cost-competitive and more easily processed without much damage to environment. The emergency of organometal halide perovskite compound CH_3_NH_3_PbI_3_ as the absorbing layer in recent years seems to be a milestone-like breakthrough for solar cell materials researches^4^,^5^,^6^. Successively broken records in the photoelectric conversion efficiency as reported on CH_3_NH_3_PbI_3_ have been made. So far recorded efficiency has reached up to 20.2% from the original 3.8%. Moreover, the production cost of such perovskite-type compounds for solar cells is low, also stimulating development of a series of other organometal halide perovskites derivatives of CH_3_NH_3_PbI_3_^7^,^8^,^9^ These events quickly have driven vigorous researches over the world.

Great amount of research in the past few years has comprehended several major issues. First, it is known that the diffusion lengths of electrons and holes in CH_3_NH_3_PbI_3_ and its derivatives are quite big^10^, thus establishing these compounds as high efficiency solar cell materials. For examples, Stranks *et al*.^11^ measured the electron and hole diffusion lengths using the photoluminescence quenching method, and found that the photon-generated carriers in CH_3_NH_3_PbI_3_ has ~100 nm in diffusion length. This length can be further enhanced for photon-generated carriers in CH_3_NH_3_PbI_3-x_Cl_x_, which is more than 1.0 μm, suggesting that the halogen family substitution or doping represents an effective and common strategy to improve the photoelectric performances. Second, the electronic structure of this compound family has been investigated. By using the light absorption test method, Grätzel *et al*. determined the binding energy to be ~30 meV for photon-generated carriers in CH_3_NH_3_PbI_3_^12^, while the value determined by Sun *et al*. is ~19 meV^13^. Such a small binding energy allows the spontaneous separation of the carriers into free electrons and holes at room temperature. Third, the electrical transport behaviors of CH_3_NH_3_PbI_3_ were found to be sensitive to processing conditions, and the carriers can be dominated by electrons or holes, as predicted by the first principles calculations. Fourth, the defect states in CH_3_NH_3_PbI_3_ were identified to impose substantial influence on the materials performances. It was reported that the defect formation energy for shallow defect states can be relatively small, while those deep defect states can have very large defect formation energy. This big difference explains why CH_3_NH_3_PbI_3_ has a big electron-hole scattering length and high open circuit voltage^14^.

Here, element substitution of CH_3_NH_3_PbI_3_ has been highly concerned since this strategy allows additional opportunities via electronic structure modulation and carrier density control to improve practical performances of these compounds for solar cell applications. Along this line, halogen element replacement and partial substitution in CH_3_NH_3_PbI_3_, as described above, have been repeatedly tried. In fact, it was reported that a Cl substitution of I in CH_3_NH_3_PbI_3_ allows the formation of CH_3_NH_3_PbI_3-x_Cl_x_^7^,^15^, whose bandgap remains similar to that of CH_3_NH_3_PbI_3_ but the positions of conduction band and valence band are modulated, leading to great promotion of diffusion and transport of carriers and thus benefiting to conversion efficiency enhancement. By using light luminescence quenching experiment, Stranks *et al*. reported a diffusion length more than 1.0 μm for photon-generated carriers in CH_3_NH_3_PbI_3-x_Cl_x_^10^. In parallel to that, Xiao *et al*. replaced a replacement of I by Br to synthesize the CH_3_NH_3_PbBr_3_^8^. It was found that the high conduction band energy level of CH_3_NH_3_PbBr_3_ can effectively enhance the open circuit voltage (*V*_*oc*_) of solar cells^16^, but the big bandgap of CH_3_NH_3_PbBr_3_ makes visible light absorption window narrow. At the same time, the large binding energy of photon-generated carriers of CH_3_NH_3_PbBr_3_ makes it difficult to spontaneously separate the carriers into free electrons and holes^15^.

In addition to the halogen substitution strategy, lattice reconstruction was believed to be effective too in improving the optical absorption properties of CH_3_NH_3_PbI_3_. Feng *et al*. successfully constructed the orthorhombic and tetragonal phases of CH_3_NH_3_PbI_3_, and calculated the optical absorptions of the two phases using the Heyd-Scuseria-Ernzerhof (HSE) screened hybrid functional (HSE06)^8^. Subsequently, the optical absorption spectra of the two phases were calculated using the quasi-particle GW correction method and multi-body interaction method^17^. Experimentally, substantial efforts have been made to probe the electronic structures and optical properties of CH_3_NH_3_PbX_3_ (X = I, Br, Cl)^8^,^10^.

Different from the above two approaches, the cationic Pb occupation in CH_3_NH_3_PbX_3_ (X = I, Cl, Br) has been thought to be irreplaceable, while the underlying reasons for this irreproducibility seems entangled to us. Either theoretical or experimental trial on replacement of Pb is still lacking. It is suggested that the upper valence band dispersion of CH_3_NH_3_PbI_3_ consists mainly of strong anti-bonding coupling of Pb-*s* and I-*p* orbitals. The strong *s-p* anti-bonding coupling makes the effective mass of holes close to that of electrons, enabling the quite big diffusion length for electron-hole pairs^12^, which is definitely unusual for solar cell applications. Nevertheless, Pb atom is a heavy metal which pollutes the environment and utilization of Pb is being strictly regulated world widely. It is immediately noted that the Bi-*s* orbital shows quite similar feature as that of the Pb-*s* orbital, making a replacement of Pb by Bi attractive for exploration. In such sense, CH_3_NH_3_BiI_3_ is likely to be a good candidate for solar cell devices, noting that Bi is non-toxic, abundant in earth, and has similar atomic properties as Pb.

In this work, our main motivation is to investigate the electronic structure and optical absorption of CH_3_NH_3_BiI_3_ as a generic substitute of CH_3_NH_3_PbI_3_ in terms of solar cell absorption properties. This motivation stems from two aspects. One is that CH_3_NH_3_BiI_3_ may exhibit broad optical absorption spectrum extending to infrared region. The other is that it is environment-friendly and cost-competitive. We employ the first-principles calculation based on the density functional theory (DFT) to study the band structure and optical absorption spectrum. The details for first-principles calculations are described in Sec. II, and the calculated results are presented and discussed in Sec. III. A brief conclusion is given in Sec. IV.

## Models and Computation Details

All calculations are performed using the Vienna *ab initio* simulation package (VASP5.2) code based on the density functional theory (DFT)^18^,^19^. The exchange and correlation potential are modeled using the generalized gradient approximation (GGA) Perdew-Becke-Erzenhof (PBE) function^20^. In standard procedure, the interaction of valence electrons with ionic core is described using the projector augmented wave (PAW) method^21^, and the wave function of the valence electron is unfolded by plane wave basis using a cutoff energy of 400 eV^14^.

**Figure 1:**
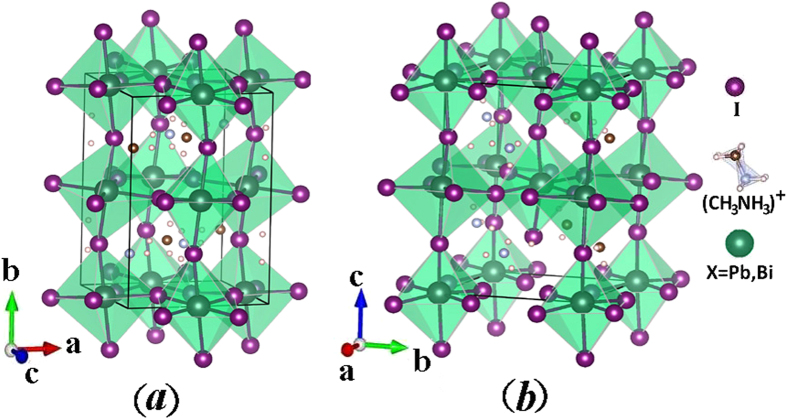
Lattice structure schematic of organometal halide perovskites materials CH_3_NH_3_XI_3_(X = Pb, B), (**a**) orthorhombic phase, (**b**) tetragonal phase.

It is noted that CH_3_NH_3_BiI_3_ is yet a proposed compound and so far no data on its synthesis and lattice structure are available. It is noted that for a stable perovskite ABX_3_, the ion radius ratio (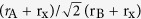) is required to be close to 1 and thus the size of A atom is much larger than that of atom B. The large-size organic ion CH_3_NH_3_^+^ can effectively stabilize this perovskite structure but does not make considerable contribution to the electronic structure of CH_3_NH_3_PbI_3_ around the band edge. For CH_3_NH_3_BiI_3_, the large-size organic ion CH_3_NH_3_^+^ which can effectively stabilize this perovskite structures does not change. In this sense, CH_3_NH_3_BiI_3_ is the same as CH_3_NH_3_PbI_3_ and should have very stable crystal structure in ambient device processing conditions. So, our calculation then starts from the structural data on reference compound CH_3_NH_3_PbI_3_. CH_3_NH_3_PbI_3_ has the orthorhombic phase and tetragonal phase, while the structure data package was successfully constructed by Feng *et al*.^8^. The structures of the two phases are shown in Fig. 1 (for simplicity, herein we denote the orthorhombic and tetragonal CH_3_NH_3_XI_3_ as O-CH_3_NH_3_XI_3_ and T-CH_3_NH_3_XI_3_ respectively). Along this line, we start from experimentally given data on CH_3_NH_3_PbI_3_ and then Bi substitution of Pb is performed, followed by sufficient structural relaxation and optimization.

The two configurations of crystal cell both contain 48 atoms. For orthorhombic and tetragonal structures, the *k*-point grids are set to 5 × 5 × 5 and 7 × 7 × 5 for the Brillouin zones respectively using the Monkhorst-Pack scheme^22^. For the optimization, the structures are fully relaxed until the Hellmann-Feynman force acting on each atom is reduced down to 10 meV/Å, and the energy convergence threshold is set as 1.0 × 10^−6 ^eV/atom. The normal generalized gradient approximation (GGA) is employed although CH_3_NH_3_PbI_3_ was reported to have the spin-orbital coupling (SOC) effect because of the strong relativistic effect of Pb^23^. However, it is known that the GGA method usually underestimates the band gap, which can offset exactly the ignored SOC effect with each other in occurrence^24^. This implies that the GGA method can give accurate bandgap of CH_3_NH_3_XI_3_, and thus the SOC will not be considered in our calculations.

## Results

### Structure optimization

In our calculations, we always take CH_3_NH_3_PbI_3_ as the reference for CH_3_NH_3_BiI_3_. The optimized structures of O-CH_3_NH_3_PbI_3_ and T-CH_3_NH_3_PbI_3_ respectively are obtained and the lattice constants are listed in Table 1, where measured and earlier predicted values on CH_3_NH_3_PbI_3_ are inserted too for comparison. It is seen that our calculated data are roughly consistent with those measured and earlier predicted values, indicating the reliability of the present computational scheme^25^,^26^. To the conventional stage, such differences are reasonably acceptable due to the relatively loose organic-inorganic hybrid structure. One sees that the Bi replacement of Pb does not induce remarkable variation of the lattice constants. The O-CH_3_NH_3_BiI_3_ has slightly tensioned *a*-axis and compressed *b*-axis with respect to the O-CH_3_NH_3_PbI_3_. For the t-CH_3_NH_3_BiI_3_, the *a*-axis is slightly tensioned but the *c*-axis is a little compressed. Considering the different definitions of lattice major axes for the two phases, the consequence of Bi replacement of Pb induces negligible variations of the lattice constants.

**Figure 2:**
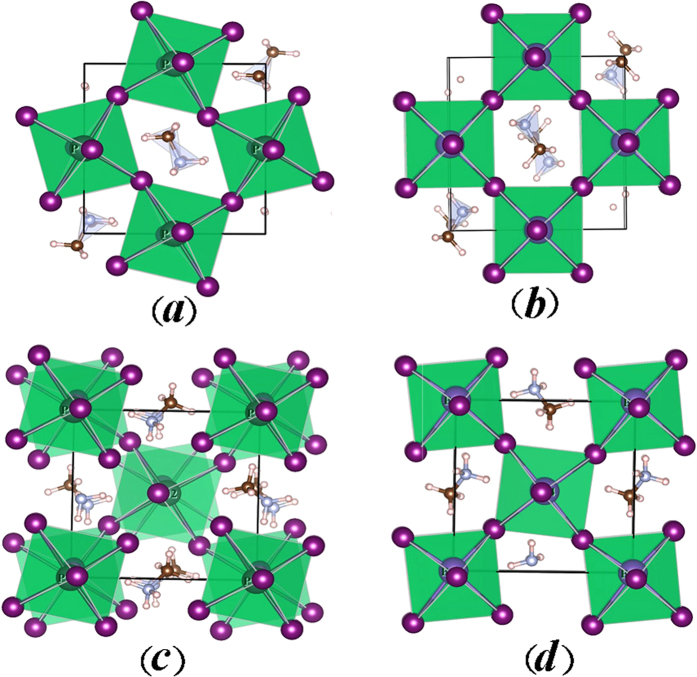
The projection diagrams of O-CH_3_NH_3_PbI_3_ in (010) plane (a), O-CH_3_NH_3_BiI_3_ in (010) plane (b), T-CH_3_NH_3_PbI_3_ in (001) plane (**c**), and T-CH_3_NH_3_BiI_3_ in (001) plane (d).

Nevertheless, no deterministic dependence of the three lattice constants on cation species at X site (Pb or Bi) can be evaluated from the data in Table 1. Alternatively, we consult to the six X-I bond lengths of the XI_6_ octahedra in order to see the differences between the two compounds and bond lengths of the XI_6_ octahedra are listed in Table 2. It is interested to notice that for O-CH_3_NH_3_BiI_3_ and T-CH_3_NH_3_BiI_3_, all the six Bi-I bonds are shorter than the corresponding Pb-I bonds of O-CH_3_NH_3_PbI_3_ and T-CH_3_NH_3_PbI_3_. In addition, the differences in XI_6_ octahedron distortion between the two compounds are distinct either. In order to see more clearly, we project the planar projections of the optimized structures (phases) and the results are presented in Fig. 2. First, the (010) plane projections of O-CH_3_NH_3_PbI_3_ and O-CH_3_NH_3_BiI_3_, presented in Fig. 2(a) and (b), respectively, show that the PbI_6_ octahedra rotate along the *b*-axis with serious distortion. The four octahedron centers within the projection plane constitute a diamond configuration. Very differently, O-CH_3_NH_3_BiI_3_ structure exhibits nearly no BiI_6_ octahedron distortion, and the four octahedron centers within the projection plane forms a square shape. The orientations of organic ion groups CH_3_NH_3_^+^ in the two compounds are different either.

The (001) plane projected configurations of T-CH_3_NH_3_PbI_3_ and T-CH_3_NH_3_BiI_3_ are plotted in Fig. 2(c) and (d) respectively. Again, the XI_6_ octahedra for both compounds have remarkable distortion which is more serious for T-CH_3_NH_3_PbI_3_. The significant difference is that the twists of the upper and lower PbI_6_ octahedra are not synchronous, but the BiI_6_ octahedra exhibit the synchronous twisting which leads to the coincidence of the upper and lower octahedron. At the same time, these octahedron distortion results in very different configurations of CH_3_NH_3_^+^ groups in the two structures, which certainly make the electronic structure quite different, to be shown below. Here, what should be mentioned here is that a more seriously distorted lattice usually favors stronger carrier localization and thus bigger bandgap. The charge transport in CH_3_NH_3_BiI_3_ is much easier due to the much weaker localization for charges in CH_3_NH_3_BiI_3_ than in CH_3_NH_3_PbI_3_. CH_3_NH_3_BiI_3_ would have better photoelectric properties than CH_3_NH_3_PbI_3_, which fits to our motivation on investigating CH_3_NH_3_BiI_3_ as a promising candidate for solar cell applications.

### Electronic structure

**Figure 3:**
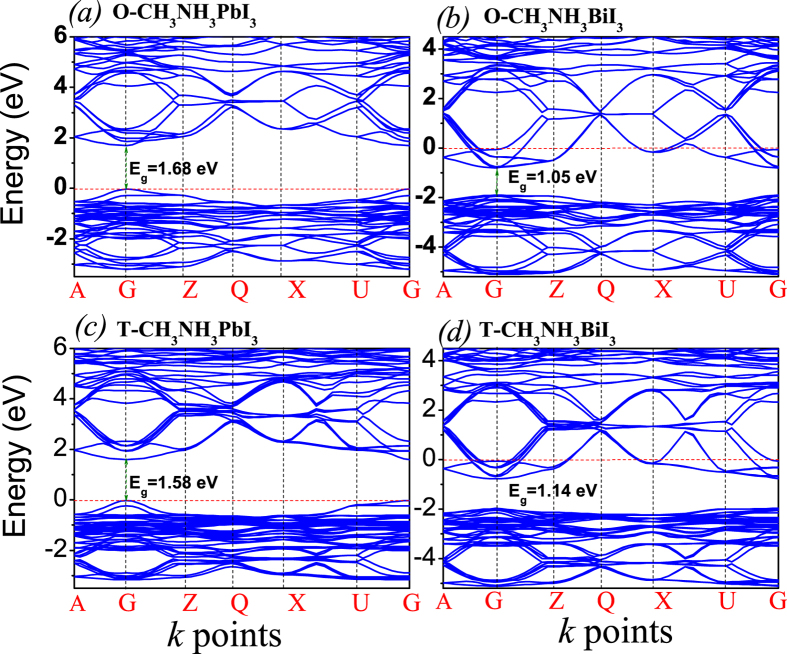
The calculated band structures of O-CH_3_NH_3_PbI_3_ (**a**), O -CH_3_NH_3_BiI_3_ (**b**), T-CH_3_NH_3_PbI_3_ (**c**), and T-CH_3_NH_3_BiI_3_ (**d**). The red dashed lines represent the Fermi level.

Given the optimized lattice structure for CH_3_NH_3_BiI_3_, one is allowed to evaluate the band structure. First, the calculated band structures for the O-phase and T-phase are plotted in Fig. 3, where the red dashed lines represent the Fermi levels in each case. The presented results can be discussed from several aspects.

First, both compounds in the o-phase are direct band gap semiconductors as seen at the G points of the Brillouin zone. The bandgap of O-CH_3_NH_3_PbI_3_ is ~1.68 eV, similar to results obtained by previous theoretical predictions^8^, while measured gap values are smaller due to the inevitable defect states. The Fermi level locates at the valence band maximum (VBM), indicating the nature of *p*-type semiconductor, while experimental measurements often reported the *n*-type semiconducting behaviors, most likely due to the defect states in samples^14^. So, lead halide perovskite CH_3_NH_3_PbI_3_ can be tuned from good p-type to good n-type by controlling the growth conditions. The bandgap of O-CH_3_NH_3_BiI_3_ is ~1.05 eV. Since Bi atom has one more electron outermost than Pb atom, the Fermi level shifts into the conduction band minimum (CBM), which makes CH_3_NH_3_BiI_3_ metallized. It is noted that such a crossing of the Fermi level with the CBM does not imply real metallization, due to the fact that there still have the energy excitation greater than the band gap energy^27^,^28^,^29^. Therefore, O-CH_3_NH_3_BiI_3_ remains to be an *n*-type degenerate semiconductor. Same to CH_3_NH_3_PbI_3_, CH_3_NH_3_BiI_3_ might be tuned from n-type to p-type by controlling the growth conditions because the thermodynamic stable range for equilibrium growth of the material cover a long shape of chemical potential region^14^. Second, as shown in Fig. 3(c) and (d) respectively, the T-CH_3_NH_3_PbI_3_ is also a *p*-type direct bandgap semiconductor with gap of ~1.58 eV, similar to earlier predictions^8^. The band structure details are similar to those of the o-phase. For T-CH_3_NH_3_BiI_3_, a direct band gap of ~1.14 eV is identified, which is 0.09 eV smaller than that of the o-phase. The Fermi level is again above the CBM, exhibiting the *n*-type degenerate semiconducting behavior.

In the overall sense, both O-CH_3_NH_3_BiI_3_ and T-CH_3_NH_3_BiI_3_ have much less lattice distortion and smaller bandgap than O-CH_3_NH_3_PbI_3_ and T-CH_3_NH_3_PbI_3_ respectively. When the band gap becomes smaller, the charge recombination chances also increases. However, a smaller band gap either allows enhanced optical excitation. In the overall sense, the number of photo-generated electron-hole pairs still increase, benefiting to the photo-voltaic performance. A preliminary prediction, consistent with the analysis on lattice structure, is that CH_3_NH_3_BiI_3_ should have better optical absorption performance than CH_3_NH_3_PbI_3_. The *n*-type carriers in CH_3_NH_3_BiI_3_ also provide alternative choice for practical applications.

**Figure 4:**
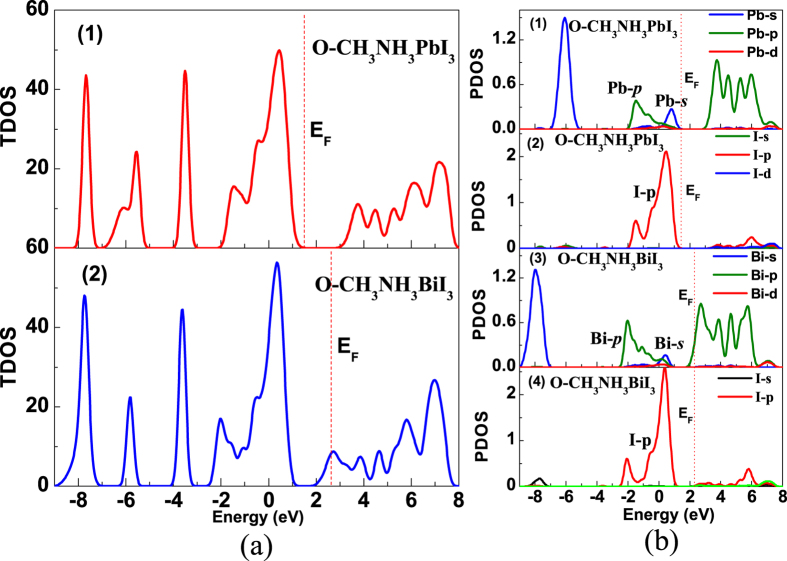
Calculated density of states of O-CH_3_NH_3_PbI_3_ and O-CH_3_NH_3_BiI_3_, (**a**) TDOS, (**b**) PDOS. The red dashed lines represent the Fermi level.

To further look into the details of electronic structure, we calculate the total density of states (TDOS) and projected density of states (PDOS) of CH_3_NH_3_PbI_3_ and CH_3_NH_3_BiI_3_ in the two phases respectively and the results are plotted in Fig. 4. The red vertical dashed line marks the Fermi level. For the o-phase, the two compounds exhibit similar TDOS profiles, as shown in Fig. 4(a). The main difference appears in the deep valence band ranging from −5.0 eV ~ −9.0 eV. It is generic to expect the similar photoelectric properties between the two compounds since electronic structure features around the conduction and valence bands are roughly the same. Figure 4(b) plots the PDOS profiles of the two compounds in the o-phase. Because the most important role of the big-size organic ion group CH_3_NH_3_^+^ is to stabilize the organic metal structure and denote an electron, one is allowed to argue nearly no contribution from the CH_3_NH_3_^+^ to the valence and conduction bands.

We first discuss the data on O-CH_3_NH_3_PbI_3_, as shown in Fig. 4(a), as a reference for subsequent discussion on O-CH_3_NH_3_PbI_3_. The O-CH_3_NH_3_PbI_3_ has a ~1.68 eV bandgap between empty Pb-*p* orbital and fully-occupied I-*p* orbital. The CBM mainly consists of Pb-*p* orbital which has nearly no coupling with I orbital, indicating the Pb-I ionic bond nature. The upper valence band mainly consists of fully-occupied Pb-*s* orbital with strong antibonding coupling with I-*p* orbital. Such band edge features were reported earlier^14^,^30^,^31^. The strong *s*-*p* antibonding coupling leads to very small effective mass of holes, comparable with electron effective mass. This is the reason why CH_3_NH_3_PbI_3_ is an ideal candidate for *p-i-n* configuration thin-film solar cells^14^.

**Figure 5:**
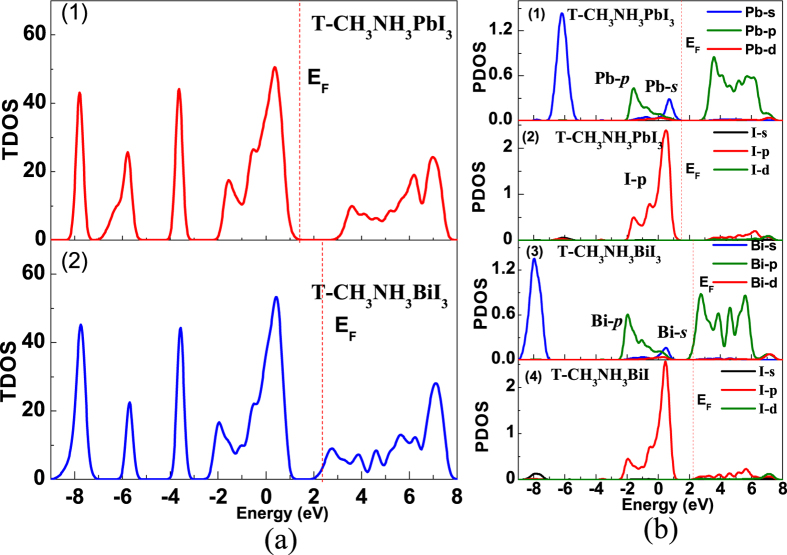
Calculated density of states of T-CH_3_NH_3_PbI_3_ and T-CH_3_NH_3_BiI_3_, (**a**) TDOS, (**b**) PDOS. The red dashed lines represent the Fermi level.

For O-CH_3_NH_3_BiI_3_, the bandgap physics shows some similarities and differences. First, the gap appears between partially-occupied Bi-*p* orbital and fully-occupied I-*p* orbital, while the CBM mainly consists of Bi-*p* orbital. The Bi-*p* orbital has either nearly no coupling with I orbital, indicating the nature of Bi-I ionic bonding. The fully-occupied Bi-*s* orbital is coupled with the I-*p* orbital in strong-antibonding form, allowing small effective mass of holes which is comparable with that of electrons in O-CH_3_NH_3_PbI_3_. In this sense, O-CH_3_NH_3_BiI_3_ can be an ideal absorbing layer for thin-film solar cells alternative to O-CH_3_NH_3_PbI_3_. Furthermore, O-CH_3_NH_3_BiI_3_ has smaller bandgap and thus enables better optical absorption performance than O-CH_3_NH_3_PbI_3_. Finally, one comes to the TDOS and PDOS of T-CH_3_NH_3_BiI_3_ while those of T-CH_3_NH_3_PbI_3_ are inserted for comparison too, as shown in Fig. 5. While delicate difference in details of the TDOS and PDOS with the results of o-phases, highly similarity of major features between two T-phases is seen immediately.

**Figure 6:**
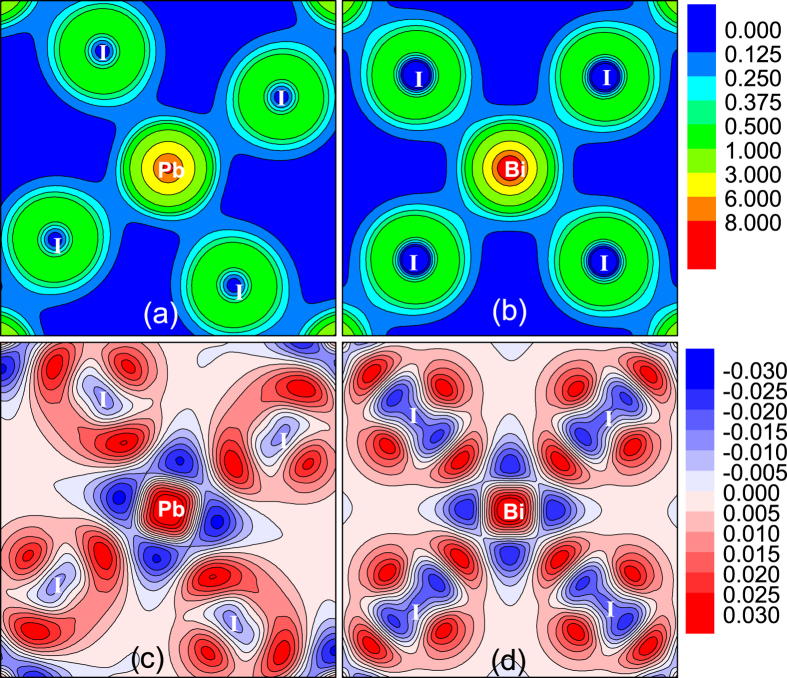
Calculated electron density O-CH_3_NH_3_PbI_3_ (**a**), O-CH_3_NH_3_BiI_3_ (**b**); the electron density difference for O-CH_3_NH_3_PbI_3_ (**c**), O-CH_3_NH_3_BiI_3_ (**d**). Contours show the values in a slice of the (010) plane. The units are electrons Å^−3^. In panels (**a**,**b**), Color from blue to red represent electron density changes from low to high. In panels (**c**,**d**), Color red (blue) represent the electron density increased (decreased).

We further consult to the calculated charge density and charge density difference for the two compounds, and the results for the O-phases are plotted in Fig. 6 illustrating the charge density on the equatorial plane of XI_6_ octahedron. First, clear octahedral distortion in O-CH_3_NH_3_PbI_3_ can be identified, consistent with earlier prediction^25^. The Pb-I ionic bonding feature is also significant. The octahedral distortion and ionic bonding in O-CH_3_NH_3_BiI_3_ are however relatively weaker. Second, the charge density in the region between Pb core and I core is less than that between Bi core and I core, and the reason is also obvious since Bi atom has one more outermost valence electron than Pb atom and the excess electron will mainly distribute on the I-*p* and Bi-*p* orbitals along the Bi-I direction. This feature enables the stronger covalent bonding character between Bi-I pair than that between Pb-I pair. Nevertheless, it should be mentioned that the Bi-I and Pb-I pair in these two materials are ionic bond dominant. A stronger covalent character between Bi-I pair only show more extensive charge spatial distribution rather than more electronic shackles. In this sense, the charges in CH_3_NH_3_BiI_3_ would show better transport performance. Third, a look at the charge density difference shown in Fig. 6(c) and (d) allows several more features. One sees that Pb loses electrons less along the Pb-I direction while the areas that Pb loses electrons most mainly appear in the four I-Pb-I sector regions divided by two neighboring I atoms and central Pb atom. I ion gets electrons most along the I-Pb direction. One also sees three strong gaining electronic regions around I ion on equatorial plane due to the torsion of PbI_6_ octahedron. Similarly, Bi loses electrons less along the Bi-I direction, and the areas that Bi loses electrons most mainly locate in the four I-Bi-I sector regions divided by two neighboring I atoms and central Bi atom. Although I atom gets electrons most along the I-Bi direction, here one sees four strong gaining electronic regions around I atom on equatorial plane, due to the fact that BiI_6_ octahedron has much less torsion.

### Optical properties

The major consequence to be expected from the above presented results is the much better optical absorption performance of CH_3_NH_3_BiI_3_ than CH_3_NH_3_PbI_3_. We use the linear response method to calculate the macroscopic optical response function described by complex dielectric function. The complex dielectric function ε(*ω*) = ε_*1*_(*ω*) + *iε*_*2*_(*ω*) contains real part ε_*1*_(*ω*) and imaginary part *ε*_*2*_(*ω*), where *ε*_*2*_(*ω*) can be obtained by calculating the momentum matrix elements between the occupied and unoccupied wave functions. Consequently, ε_*1*_(*ω*) is calculated from *ε*_*2*_(*ω*) using the Kramer–Kronig relationship^32^. It is noted that the optical absorption spectrum is characterized by the optical constant which is related with the dielectric function 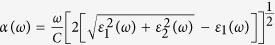, where *α* is the absorption coefficient, *C* the speed of light, *ω* the circular frequency.

**Figure 7:**
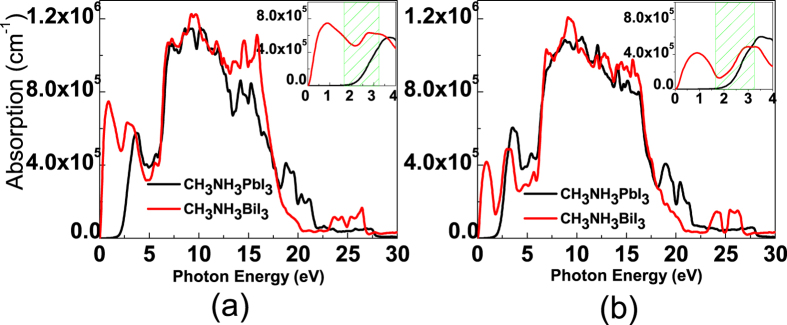
Calculated optical absorption spectrum of O-CH_3_NH_3_XI_3_(Pb Bi), (**a**), and T-CH_3_NH_3_XI_3_(Pb,Bi) (**b**).

The calculated optical absorption spectrum for the O-phase and T-phase of CH_3_NH_3_PbI_3_ and CH_3_NH_3_BiI_3_ are plotted in Fig. 7(a) and (b), noting that visible light energy appears in 1.64 eV~3.19 eV, as marked out in the inset using green oblique lines. No matter what phase it is, one sees two major consequences. First, for CH_3_NH_3_BiI_3_, the long-wavelength boundary is substantially extended toward the infrared region, in which the optical absorption is significant as seen more clearly in the two insets. Second, CH_3_NH_3_BiI_3_ has even better absorption performance in the visible region than CH_3_NH_3_PbI_3_. The two characters of CH_3_NH_3_BiI_3_ suggest that using Bi to replace Pb in CH_3_NH_3_PbI_3_ would be a highly favored strategy, while CH_3_NH_3_BiI_3_ is expected to be a promising candidate for highly efficient absorption media for solar cell devices.

## Discussions

In this paper, we have carefully calculated the electronic structures and optical properties of organometal halide perovskites materials CH_3_NH_3_XI_3_(X = Pb, Bi) using the first principle calculation. Our results indicate that CH_3_NH_3_PbI_3_ is a direct band gap semiconductor. The strong antibonding coupling state of Pb-*s* orbital and I-*p* orbital compose of the top of the valence band distribution. For CH_3_NH_3_BiI_3_, the band gap is narrow with respect to CH_3_NH_3_PbI_3_. The strong antibonding coupling states of Bi-*s* orbital and I-*p* orbital compose of the top of the valence band distribution of CH_3_NH_3_BiI_3_, and the band edge structure is similar to that of CH_3_NH_3_PbI_3_. The optical data show that CH_3_NH_3_BiI_3_ has stronger visible light absorption than CH_3_NH_3_PbI_3_ and infrared absorption is predicted. These similar electronic structures and better spectral absorption indicate that CH_3_NH_3_BiI_3_ system is likely to be a good candidate for solar battery.

## Additional Information

**How to cite this article**: Zhu, H. X. and Liu, J.-M. Electronic structure of organometal halide perovskite CH_3_NH_3_BiI_3_ and optical absorption extending to infrared region. *Sci. Rep*. **6**, 37425; doi: 10.1038/srep37425 (2016).

**Publisher's note**: Springer Nature remains neutral with regard to jurisdictional claims in published maps and institutional affiliations.

## Figures and Tables

**Table 1:** Lattice structure optimization and lattice constants evaluation (O- & T- for orthorhombic & tetragonal structures respectively).

Materials	Obtained values in this work			Measured (exp) and obtained (theo) values in literature			
	*a* (Å)	*b* (Å)	*c* (Å)	*a* (Å)	*b* (Å)	*c* (Å)	Ref.
O-CH_3_NH_3_PbI_3_	8.546	12.856	9.043	8.836	12.580	8.555	25, 26
				8.376	12.247	9.017	8
T-CH_3_NH_3_PbI_3_	8.834	8.789	12.978	8.80	8.80	12.69	33
				9.06	8.77	12.91	34
				8.94	8.94	12.98	
O-CH_3_NH_3_BiI_3_	8.832	12.679	9.002				
T-CH_3_NH_3_BiI_3_	8.985	8.798	12.682				

**Table 2:** The six X-I bond lengths corresponding to the XI_6_ octahedra (X = Pb, Bi).

Materials	X-I bond lengths in the equatorial plane				Two X-I apical bond lengths	
	X-I_1_ (Å)	X-I_2_ (Å)	X-I_3_ (Å)	X-I_4_ (Å)	X-I_5_ (Å)	X-I_6_ (Å)
O-CH_3_NH_3_PbI_3_	3.21159	3.22928	3.21159	3.22928	3.23676	3.23676
T-CH_3_NH_3_PbI_3_	3.23876	3.19168	3.18230	3.21603	3.25869	3.28000
O-CH_3_NH_3_BiI_3_	3.14847	3.1614	3.14847	3.16142	3.17172	3.17172
T-CH_3_NH_3_BiI_3_	3.18504	3.16592	3.16248	3.15525	3.17870	3.17313
